# Supplementary material for: Role of PDGFRA+ cells and a CD55+ PDGFRALo fraction in the gastric mesenchymal niche
Source: Nat Commun. 2023 Dec 2;14:7978. doi: 10.1038/s41467-023-43619-y (PMC10693581; doi:10.1038/s41467-023-43619-y)
Supplement: Supplementary file 1 — Supplementary Information [file 41467_2023_43619_MOESM1_ESM.pdf]

Supplementary Information

Role of PDGFRA$^+$ cells and a CD55$^+$ PDGFRA^{Lo} fraction in the gastric mesenchymal niche

Elisa Manieri[1,2], Guodong Tie[1], Ermanno Malagola[3], Davide Seruggia[4,5,6], Shariq Madha[1], Adrianna Maglieri[1], Kun Huang[7], Yuko Fujiwara[3], Kevin Zhang[3], Stuart H. Orkin[3,8,9], Timothy C. Wang[3], Ruiyang He[1], Neil McCarthy[1,2], Ramesh A. Shivdasani[1,2,9,*]

[1]Department of Medical Oncology and Center for Functional Cancer Epigenetics, Dana-Farber Cancer Institute, Boston, MA 02215, USA; [2]Department of Medicine, Harvard Medical School, Boston, MA 02115, USA; [3]Division of Digestive and Liver Diseases, Department of Medicine and Irving Cancer Research Center, Columbia University Medical Center, New York, NY 10032, USA; [4]Department of Hematology, Boston Children's Hospital, Boston, MA 02115, USA; [5]St. Anna Children's Cancer Research Institute, Vienna, Austria; [6]CeMM Research Center for Molecular Medicine of the Austrian Academy of Sciences, Vienna, Austria; [7]Molecular Imaging Core and Department of Cancer Immunology and Virology, Dana-Farber Cancer Institute, Boston, MA 02215, USA; [8]Howard Hughes Medical Institute, Boston, MA 02115, USA; [9]Harvard Stem Cell Institute, Cambridge, MA 02138, USA.

*Corresponding author: Ramesh A. Shivdasani, MD, PhD
Dana-Farber Cancer Institute
450 Brookline Avenue, Boston, MA 02215, USA
ramesh_shivdasani@dfci.harvard.edu

a

Marker	Cell type/Structure
CD31	Endothelial cells
SMA1	Smooth muscle cells
LAM	Basal lamina
PDGFRA	PDGFRA^{+} cells
GSII	Neck/Basal mucous cells
UEAI	Foveolar cells
Tnfrsf19	Chief cells (corpus)
Atp4a	Parietal cells (corpus)
Atp4b	Parietal cells (corpus)
Pgc	Chief cells (corpus)
Gif	Chief cells (corpus)
Axin2	Antrum proliferative cells
Cd44	Antrum proliferative cells
Gast	Endocrine G-cells (antrum)
ChgA	Neuro-endocrine cells (antrum)
ChgB	Neuro-endocrine cells (antrum)
Cd34	PDGFRA^{Lo} cells
MUC5AC	Foveolar cells
GAST	Endocrine G-cells
SST	Endocrine Somatostatin-producing cells
Ki67	Proliferative cells

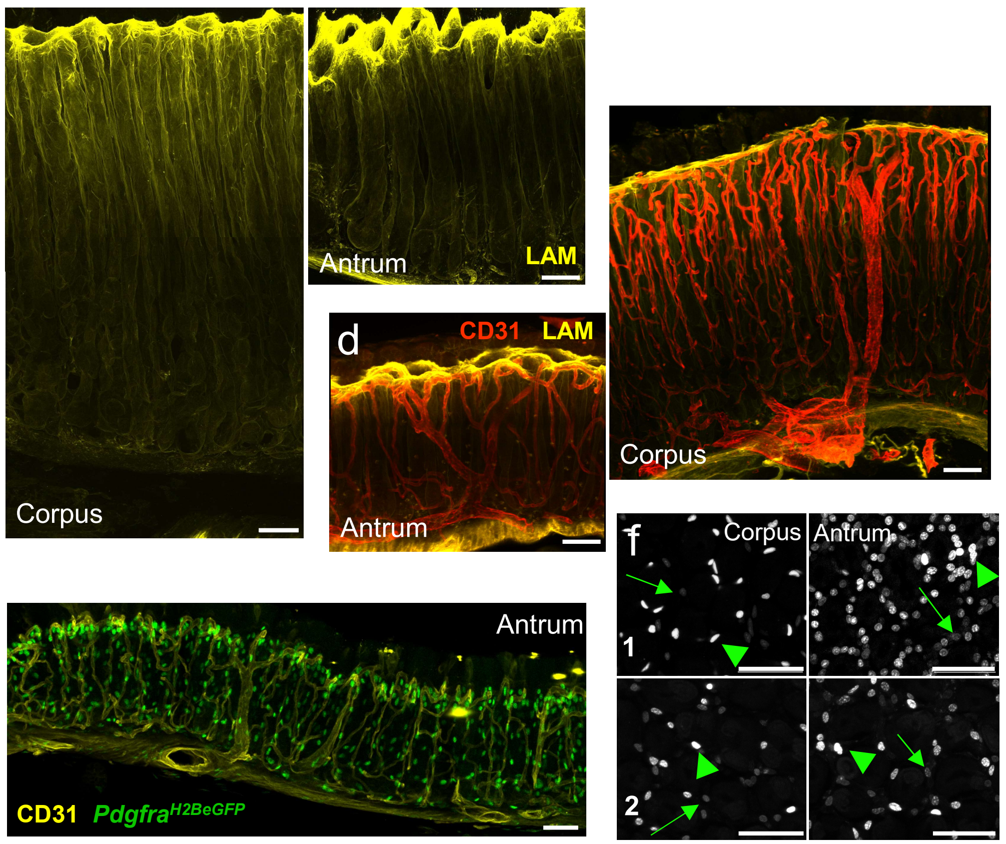

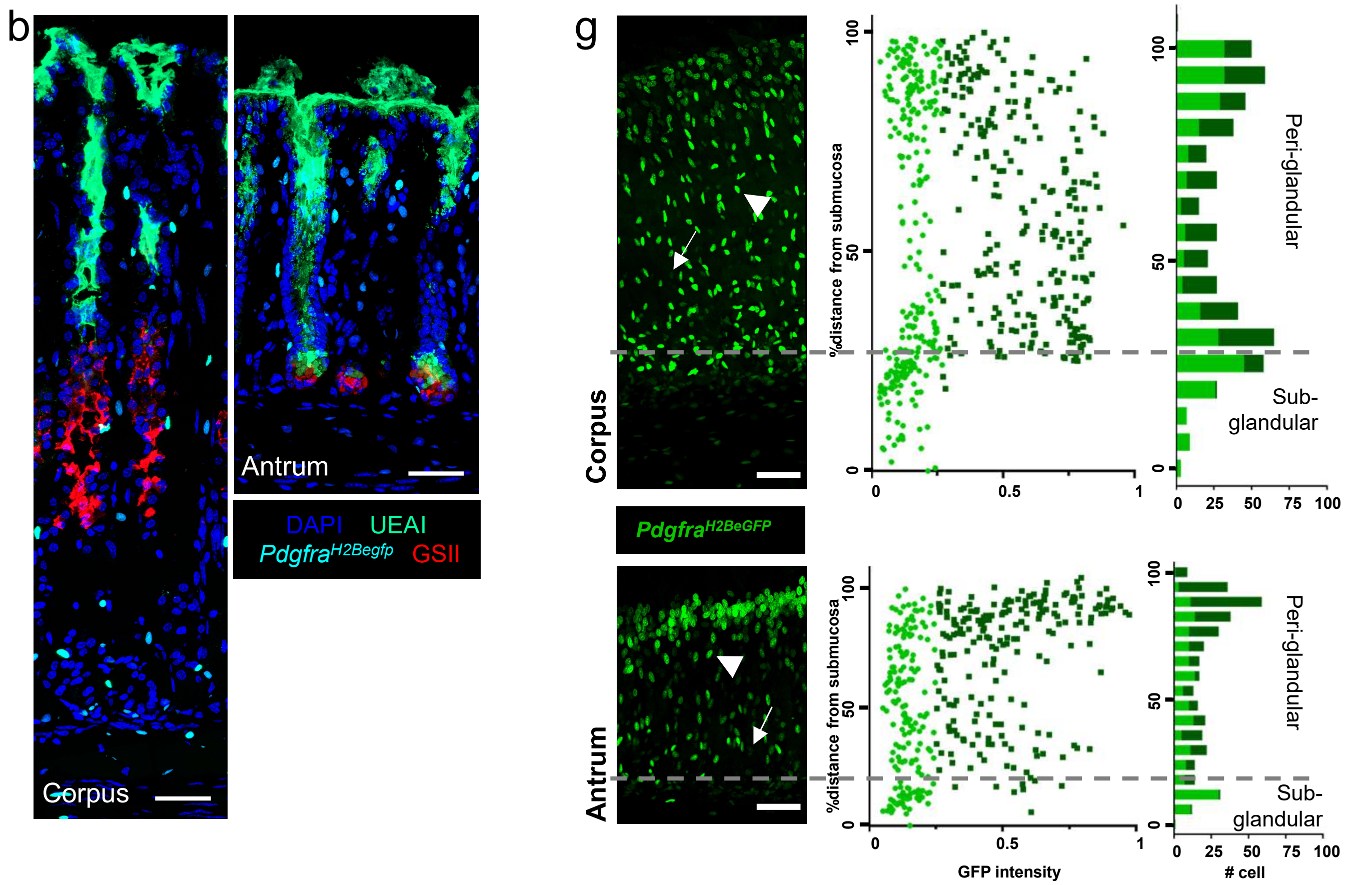

Supplementary Figure 1. Structure and organization of gastric corpus and antral mesenchyme.

(a) Table summarizing the markers used throughout the study to identify different structures or cell types.

(b) UEA-I and GS-II staining of *Pdgfra*^{H2BeGFP} corpus (left) and antrum (right) sections, highlighting the distribution of mucous producing cells, respectively foveolar cells (UEA-I, neon green) and base/neck mucous cells (GS-II, red). Scale bars represent 50 µm.

(c) Whole-mount 3D rendering of Laminin antibody-stained corpus (left) and antral (right) tissue, highlighting the epithelium-mesenchyme interface and gland foveolae (pits). Scale bars represent 50 µm.

(d) Whole-mount 3D rendering of antrum (left) and corpus (right) tissue, showing capillary (CD31, red) dimensions and organization extending from sub-mucosal arterioles. Capillaries branch along the whole length of corpus glands, while antral capillaries branch only near pits. Yellow, Laminin immunostaining. Scale bars represent 50 µm.

(e) Whole-mount 3D rendering of *Pdgfra*^{H2BeGFP} antral glands, showing vascular (CD31, yellow) organization and high density of PDGFRA^{Hi} (green) SEMFs across the tissue. Scale bar represents 50 µm.

(f) Grayscale image of GFP signals from the gland cross-sections. These images highlight differential GFP expression in PDGFRA^{Hi} (arrowheads) and PDGFRA^{Lo} (arrows) cells. Scale bars represent 50 µm.

(g) Whole-mount 3D rendering of *Pdgfra*^{H2BeGFP} corpus (top) and antral (bottom) glands. PDGFRA^{Hi} (arrowheads) and PDGFRA^{Lo} (arrows) cells are indicated. Scale bars represent 50 µm. Right: GFP signals quantified along gland lengths show highest concentrations of PDGFRA^{Lo} in the sub-glandular area of both regions, lower concentration at corpus isthmus/neck, and high concentration of PDGFRA^{Hi} at antral pits.

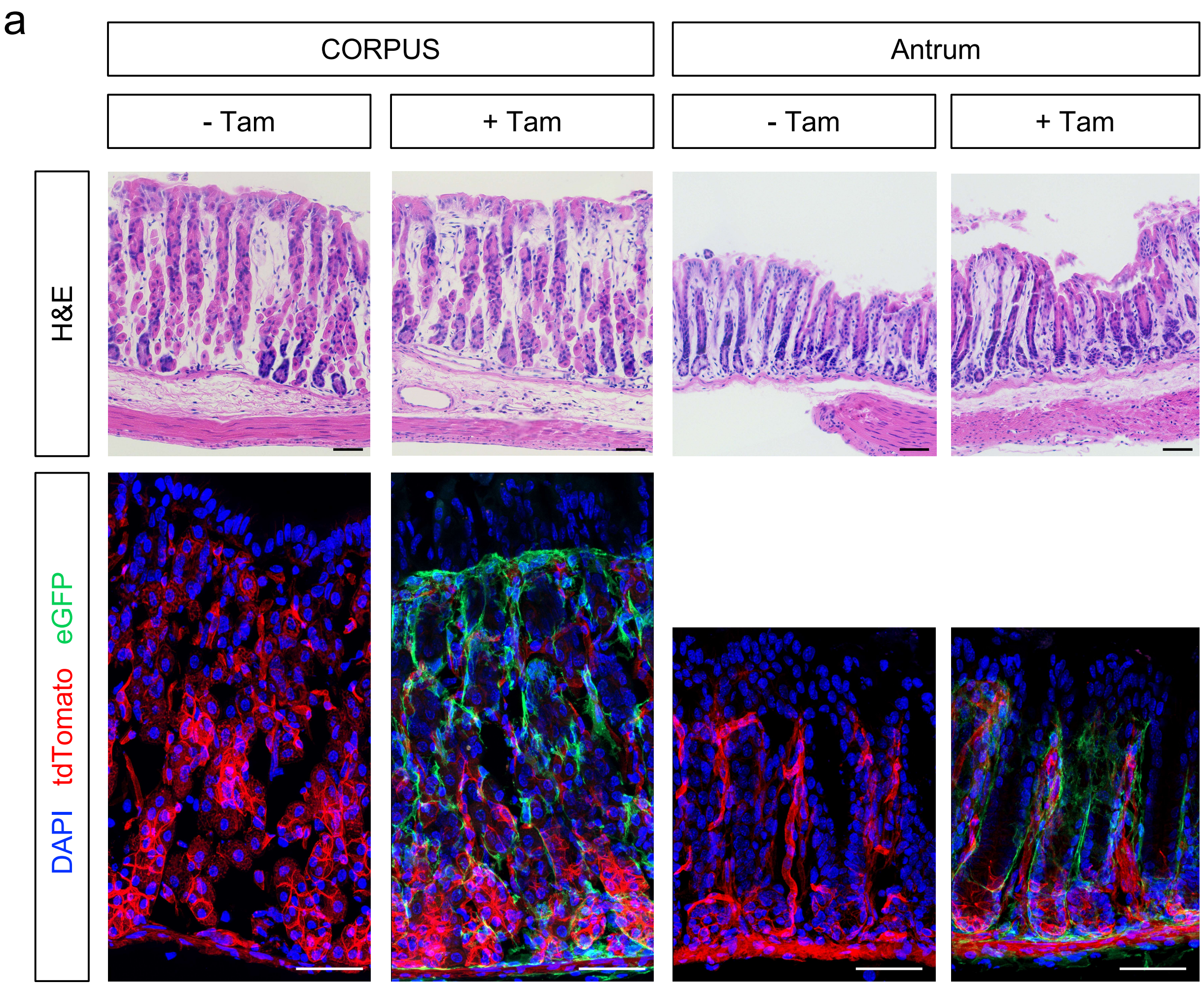
a
CORPUS
Antrum
- Tam
+ Tam
- Tam
+ Tam
H&E
DAPI tdTomato eGFP

Supplementary Figure 2. *Pdgfra*-expressing cells in corpus and antrum mesenchyme.

(a) Histology (H&E) and immunofluorescence imaging of corpus (left) and antrum (right) from *Pdgfra*$^{Cre(ER\text{-}T2)}$;*Rosa26*$^{mT/mG}$ mice with or without tamoxifen treatment. H&E staining shows the absence of tamoxifen-induced toxicity in the stomach. Immunofluorescence pictures show that every cell expressed TdTomato before Cre-Recombinase activation (-Tam), while *Pdgfra*-expressing cells stained green after tamoxifen treatment. Scale bars represent 50 μm.

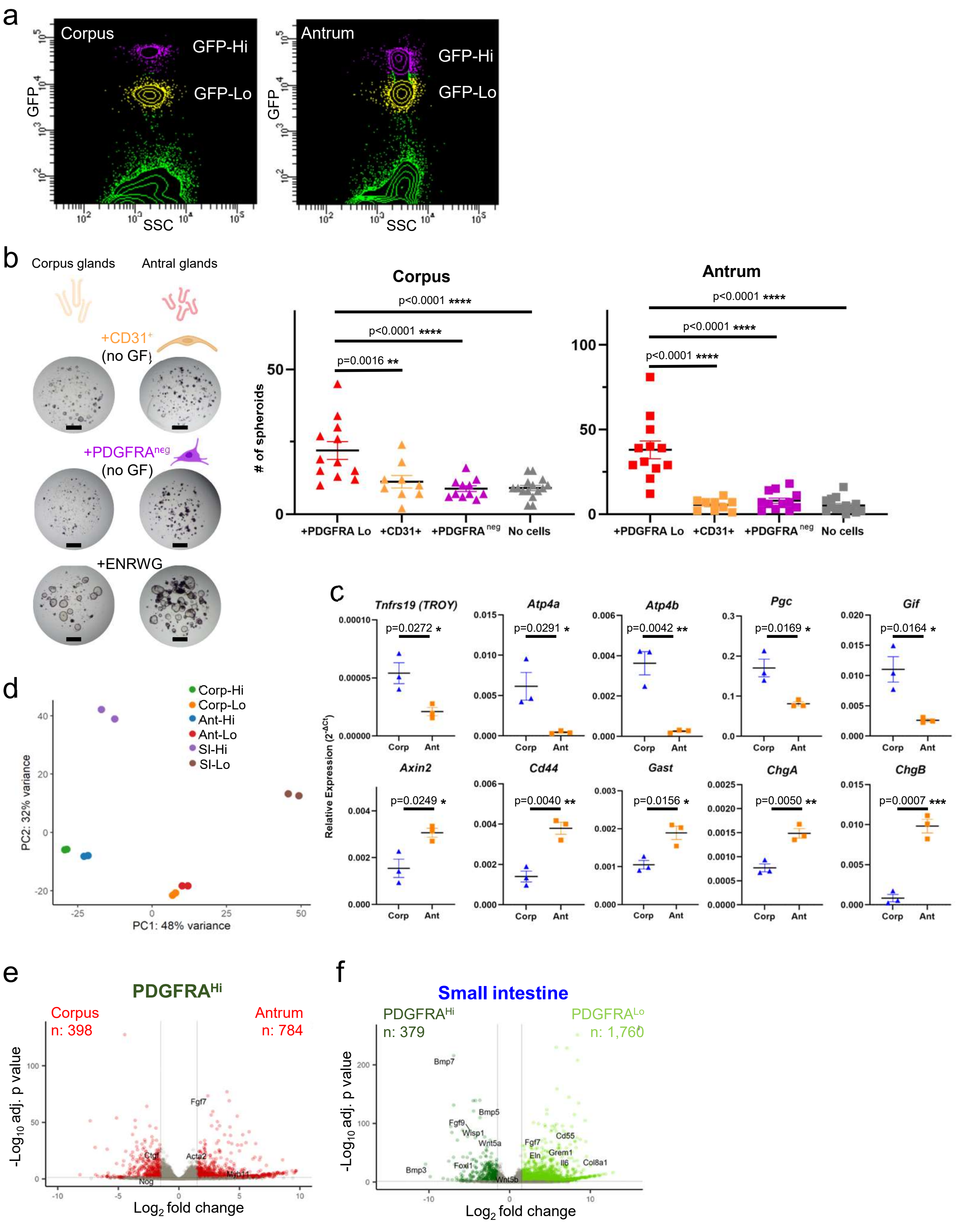
a
Corpus
GFP-Hi
GFP-Lo
GFP
SSC
Antrum
GFP-Hi
GFP-Lo
b
Corpus glands
Antral glands
+CD31+
(no GF)
+PDGFRAneg
(no GF)
+ENRWG
Corpus
p<0.0001 ****
p<0.0001 ****
p=0.0016 **
# of spheroids
+PDGFRA Lo
+CD31+
+PDGFRAneg
No cells
Antrum
p<0.0001 ****
p<0.0001 ****
p<0.0001 ****
c
Tnfrs19 (TROY)
p=0.0272 *
Atp4a
p=0.0291 *
Atp4b
p=0.0042 **
Pgc
p=0.0169 *
Gif
p=0.0164 *
Relative Expression (2-ΔCt)
Axin2
p=0.0249 *
Cd44
p=0.0040 **
Gast
p=0.0156 *
ChgA
p=0.0050 **
ChgB
p=0.0007 ***
Corp
Ant
d
Corp-Hi
Corp-Lo
Ant-Hi
Ant-Lo
SI-Hi
SI-Lo
PC2: 32% variance
PC1: 48% variance
e
PDGFRAHi
Corpus
n: 398
Antrum
n: 784
-Log10 adj. p value
Log2 fold change
Fgf7
Ctgf
Acta2
Nog
Myh11
f
Small intestine
PDGFRAHi
n: 379
PDGFRALo
n: 1,760
Bmp7
Bmp5
Fgf9
Wisp1
Wnt5a
Bmp3
Foxl1
Wnt5b
Fgf7
Eln
Cd55
Grem1
Il6
Col8a1

Supplementary Figure 3. Properties and dependencies of gastric corpus and antral spheroids and differential expression among PDGFRA^{Hi} (SEMFs) and PDGFRA^{Lo} cells.

(a) Separation of PDGFRA^{Hi} from PDGFRA^{Lo} cells from whole *Pdgfra*^{H2BeGFP} corpus (top) and antral (bottom) mesenchyme by GFP flow cytometry. Gating strategy reported in Supplementary figure 9b.

(b) Left: Matrigel co-culture of glands from different gastric segments with CD31^{+} endothelial cells or PDGFRA^{neg} cells isolated from the same segment do not induce spheroids formation in the absence of rNOG and RSPO1. Gastric glands growth in ENRWG complete medium are included as a control. Scale bars 400 µm. Right: Quantification of spheroid formation with PDGFRA^{Lo}, CD31^{+} endothelial cells or PDGFRA^{neg} cells (Corpus: +PDGFRA Lo n=12, +CD31+ n=9, +PDGFRA^{neg} n=11, No cells n=15; Antrum: +PDGFRA Lo n=12, +CD31+ n=9, +PDGFRA^{neg} n=12, No cells n=14). Bars represent mean ±SEM values. Significance of differences determined by ordinary one-way ANOVA coupled with Dunnett's multiple comparison test. Source data are provided as a Source Data file. Gating strategy for cell isolation reported in Supplementary Fig. 9b,c.

(c) qRT-PCR analysis of corpus and antral epithelial cell markers (relative to *Gapdh*) in spheroids cultured for 4 days in complete ENRWG medium. Spheroids show the expected differences in expression of region-specific marker genes (corpus, blue; antrum, orange). Significance of differences determined by two-tailed unpaired Student's t-test statistical analysis. n=3 independent experiments. Source data are provided as a Source Data file.

(d) Principal components analysis (PCA) of duplicate bulk RNA-seq libraries from the indicated cell types, showing high concordance between replicates. SI, small intestine; Corp, corpus; Ant, antrum; Hi: PDGFRA^{Hi} cells (SEMFs); Lo: PDGFRA^{Lo} cells.

(e,f) Genes differentially expressed (q <0.05; $\log_2$ fold-difference >1.5) between corpus and antrum PDGFRA^{Hi} (**e**), and between PDGFRA^{Hi} and PDGFRA^{Lo} cells isolated from the SI (**f**). Selected genes are labeled. Significance of differences determined by DESeq2 default settings (Wald test).

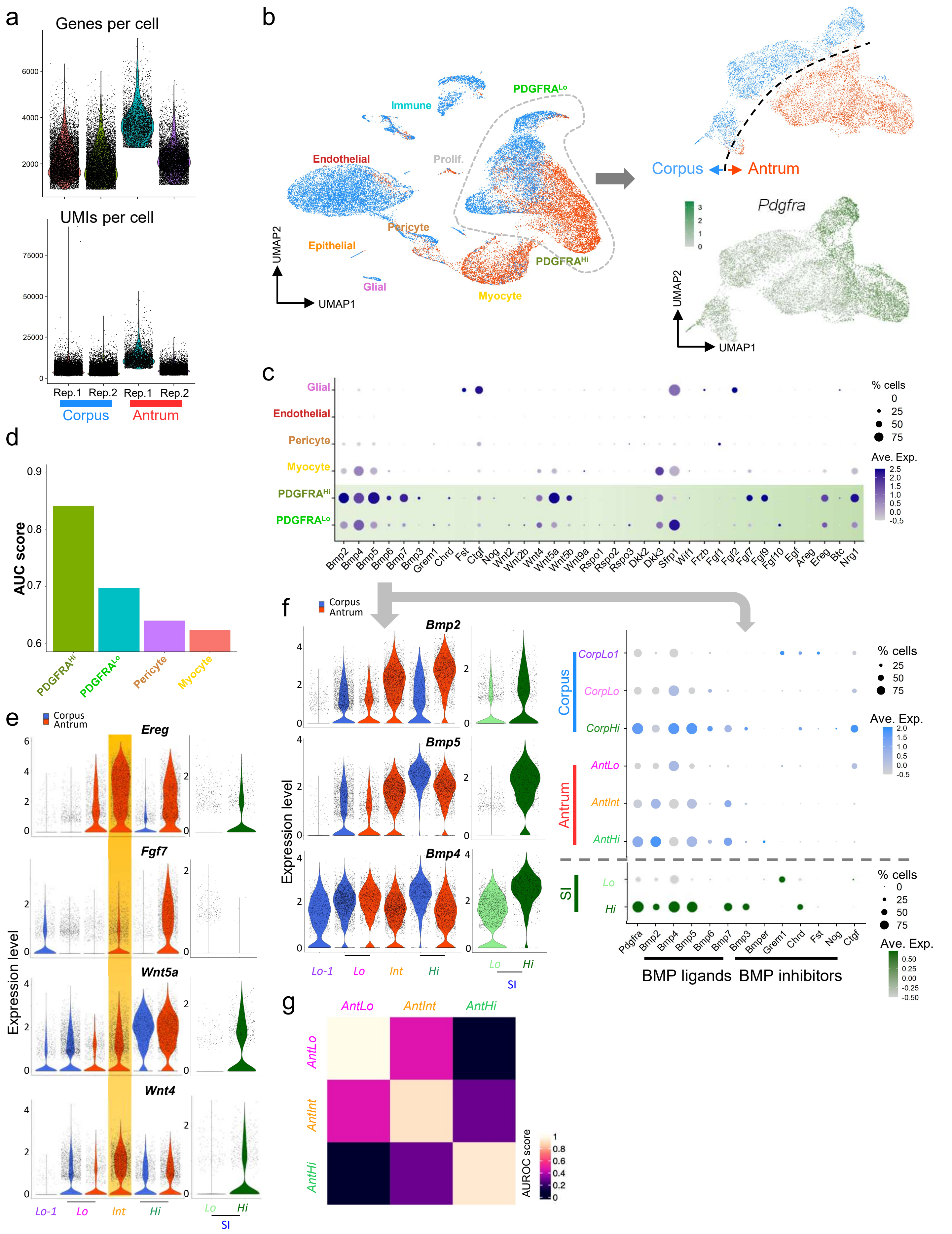
a
Genes per cell
UMIs per cell
Rep.1
Rep.2
Rep.1
Rep.2
Corpus
Antrum
b
Immune
PDGFRA^Lo
Endothelial
Prolif.
Pericyte
Epithelial
Glial
PDGFRA^Hi
Myocyte
UMAP1
UMAP2
Corpus
Antrum
Pdgfra
c
Glial
Endothelial
Pericyte
Myocyte
PDGFRA^Hi
PDGFRA^Lo
% cells
Ave. Exp.
d
AUC score
PDGFRA^Hi
PDGFRA^Lo
Pericyte
Myocyte
e
Corpus
Antrum
Ereg
Fgf7
Wnt5a
Wnt4
Expression level
Lo-1
Lo
Int
Hi
SI
f
Bmp2
Bmp5
Bmp4
CorpLo1
CorpLo
CorpHi
AntLo
AntInt
AntHi
Corpus
Antrum
SI
BMP ligands
BMP inhibitors
g
AntLo
AntInt
AntHi
AUROC score

Supplementary Figure 4. Gastric corpus and antral mesenchymal transcripts at single-cell resolution.

(a) Parameters of data quality from replicate samples (Rep.) of single mesenchymal cells. UMI: unique molecular identifier.

(b) Left: Delineation of mesenchymal populations by Uniform manifold approximation and projection (UMAP) of 20,624 corpus (blue dots) and 13,821 antral (orange dots) cells resolved by differential gene expression. mRNA profiles of all cell types, except *Pdgfra*-expressing cells, from the two gastric segments overlap extensively. Right: UMAP plot of these *Pdgfra*$^{+}$ cells extracted from the data on whole mesenchyme separate objectively according to their corpus (6,594 cells) or antral (5,896 cells) origins. Projection of *Pdgfra* transcript density onto the UMAP plot shows *Pdgfra*^{Hi} cells toward the right, *Pdgfra*^{Lo} cells toward the left, and a previously unapparent subset of antral cells with intermediate *Pdgfra* mRNA levels (AntInt, see Figure 3).

(c) Relative expression of selected BMP, Wnt, FGF, and EGF pathway genes in all mesenchymal populations identified by scRNA-seq. Circle diameters represent the fraction of cells expressing a gene and fill shading, from dark to light purple, represents the normalized average expression in each population. These signaling genes express principally in PDGFRA^{+} cells.

(d) AUGUR analysis on mesenchymal populations with at least 200 cells for both regions. PDGFRA^{Hi} and PDGFRA^{Lo} result in the highest AUC (area under the curve) score, indicating greater transcriptional differences in these populations between corpus and antrum and affirming that the differences we report are biological rather than technical effects.

(e) Relative expression of *Ereg, Fgf7* and *Wnt5a* genes in the identified *Pdgfra*-expressing populations and the corresponding SI cells. Blue, corpus; red, antrum; green, SI.

(f) Left: Relative expression of *Bmp* ligand genes in the identified *Pdgfra*-expressing populations and the corresponding SI cells. Blue, corpus; red, antrum; green, SI. Right: Relative expression of selected BMP and BMPi genes in gastric and SI PDGFRA^{+} populations. Circle sizes represent the percentage of expressing cells and fill colors represent normalized average expression. Gastric PDGFRA^{+} cells provide a higher BMP tone than in the SI.

(g) MetaNeighbour AUROC (area under the receiver operating characteristic) analysis shows AntInt population is more similar to AntLo than to AntHi.

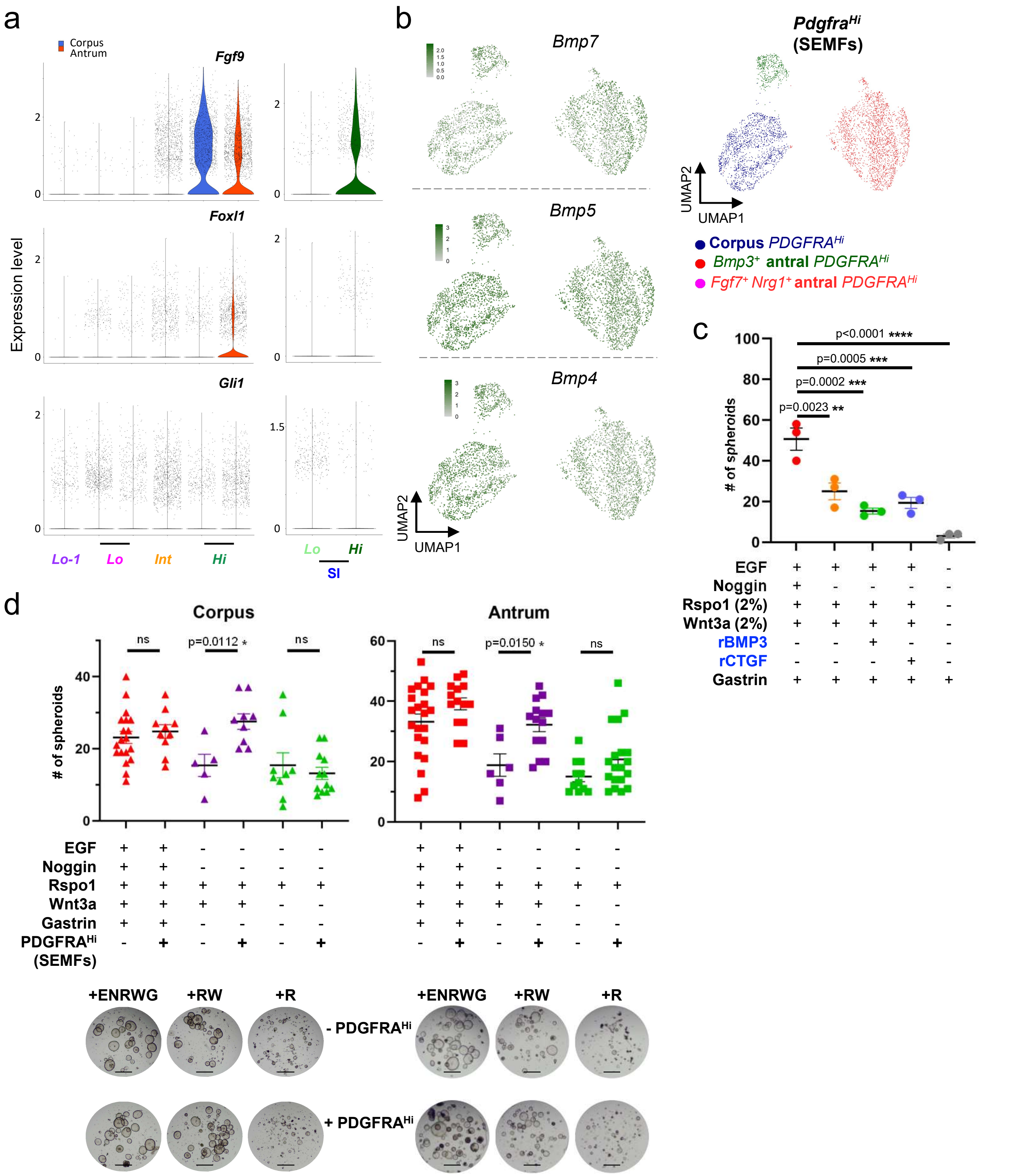
a
Corpus
Antrum
Fgf9
Foxl1
Gli1
Expression level
Lo-1
Lo
Int
Hi
Lo
Hi
SI
b
Bmp7
Bmp5
Bmp4
UMAP2
UMAP1
PdgfraHi
(SEMFs)
Corpus PDGFRAHi
Bmp3+ antral PDGFRAHi
Fgf7+ Nrg1+ antral PDGFRAHi
c
p<0.0001 ****
p=0.0005 ***
p=0.0002 ***
p=0.0023 **
# of spheroids
EGF
Noggin
Rspo1 (2%)
Wnt3a (2%)
rBMP3
rCTGF
Gastrin
d
Corpus
Antrum
ns
p=0.0112 *
p=0.0150 *
# of spheroids
EGF
Noggin
Rspo1
Wnt3a
Gastrin
PDGFRAHi
(SEMFs)
+ENRWG
+RW
+R
- PDGFRAHi
+ PDGFRAHi

Supplementary Figure 5. Characteristics of regionally distinct gastric SEMF cells.

(a) Relative expression of *Fgf9, Foxl1* and *Gli1* genes in the identified *Pdgfra*-expressing populations and the corresponding SI cells. *Fgf9* expression distinguish SEMFs from corpus and SI. Blue, corpus; red, antrum; green, SI.

(b) UMAP plots of gastric PDGFRA^{Hi} cells extracted from scRNA analysis of whole mesenchyme, resolved into 3 populations: homogeneous corpus cells and two antral cell types, one expressing high *Bmp3* and the other expressing high *Fgf7* and *Nrg1*. Other BMP genes, e.g., *Bmp7, Bmp5* and *Bmp4*, express uniformly among the PDGFRA^{Hi} populations (SEMFs – see also Figure 4b).

(c) Quantitation of rBMP3 and rCTGF effects on antral spheroid formation. Antral glands were treated with these recombinant factors in addition to media with reduced concentrations (2%) of RSPO1 and AFAMIN/Wnt3a and lacking rNOG. Optimized for sensitive detection of BMPi effects, these conditions, failed to elicit such activity from rBMP3 or rCTGF (n= 3). Bars represent mean ±SEM values. Significance of differences was determined by ordinary one-way ANOVA coupled with Tukey's multiple comparison test. Source data are provided as a Source Data file.

(d) Matrigel co-culture of corpus (left) and antral (right) glands with unfractionated PDGFRA^{Hi} cells, supplemented with complete medium (ENRWG), Rspo1 and Wnt3a medium (RW) or addition of Rspo1 to minimal medium (R). Glands cultured in the respective media with no cell addition serve as a control (Corpus: +ENRW No cells n=19, +ENRW + PDGFRA Hi n=10, +RW No Cells n=5, +RW + PDGFRA Hi n=9, +R No Cells n=9, +R + PDGFRA Hi n=12; Antrum: +ENRW No cells n=22, +ENRW + PDGFRA Hi n=14, +RW No Cells n=6, +RW + PDGFRA Hi n=14, +R No Cells n=11, +R + PDGFRA Hi n=19). In the presence of growth factors, PDGFRA^{Hi} did not affect spheroid formation. Scale bars 400 μm. Results are quantified above. Bars represent mean ±SEM values. Significance of differences was determined by ordinary one-way ANOVA coupled with Sidak's multiple comparison test. ns: not significant.

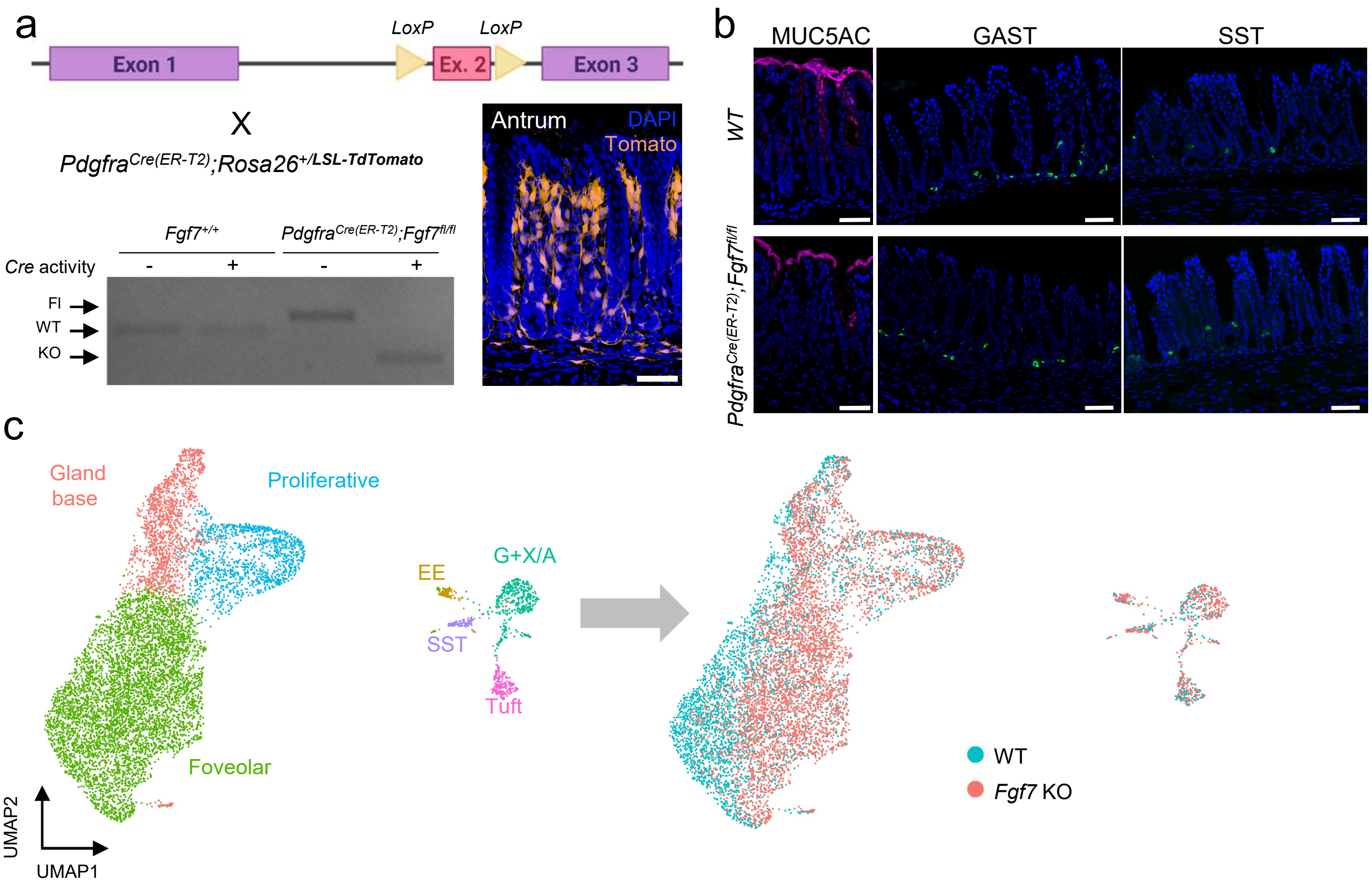
a
LoxP
LoxP
Exon 1
Ex. 2
Exon 3
X
PdgfraCre(ER-T2);Rosa26+/LSL-TdTomato
Fgf7+/+
PdgfraCre(ER-T2);Fgf7fl/fl
Cre activity
-
+
-
+
Fl
WT
KO
Antrum
DAPI
Tomato
b
MUC5AC
GAST
SST
WT
PdgfraCre(ER-T2);Fgf7fl/fl
c
Gland base
Proliferative
Foveolar
EE
G+X/A
SST
Tuft
UMAP2
UMAP1
WT
Fgf7 KO

Supplementary Figure 6. Genetic disruption of *Fgf7* in PDGFRA^{+} cells.

(a) Schematic representation of an engineered floxed *Fgf7*$^{fl/fl}$ allele. The agarose gel below resolves genotyping PCR products in mesenchymal cells isolated from *Fgf7*$^{+/+}$ (wild-type controls) and *Pdgfra*$^{Cre(ER\text{-}T2)}$;*Fgf7*$^{fl/fl}$ animals with (2 mg tamoxifen injected i.p. on 4 consecutive days) or without activation of Cre recombinase. A representative antral tissue section (n= 3 mice) from a *Pdgfra*$^{Cre(ER\text{-}T2)}$;*Rosa26*$^{LSL\text{-}tdTomato}$ mouse shows abundant Cre activity in PDGFRA^{Hi} cells after tamoxifen exposure (see also Figure 1e and Supplementary Movies 5 and 6). Source data are provided as a Source Data file.

(b) Immunostained antral tissues from wild-type (WT) and *Pdgfra*$^{Cre(ER\text{-}T2)}$;*Fgf7*$^{fl/fl}$ mice reveal no discernible impact of mesenchymal FGF7 deficiency on MUC5AC expression and secretion or on the number and distribution of enteroendocrine GAST- and SST-producing cells. n= 2-3 independent experiments for each marker. Scale bars 50 µm.

(c) UMAP clustering of antral epithelial cells from RNA profiling of 3,885 wild-type (WT) and 5,595 mesenchymal *Fgf7*-deficient mice 90 days after *Pdgfra*$^{Cre(ER\text{-}T2)}$ animals were treated with tamoxifen. Antral epithelium is essentially unchanged. Although WT and mutant foveolar cells did not overlap perfectly, differences in gene expression (listed in Supplementary Table 3) were subtle.

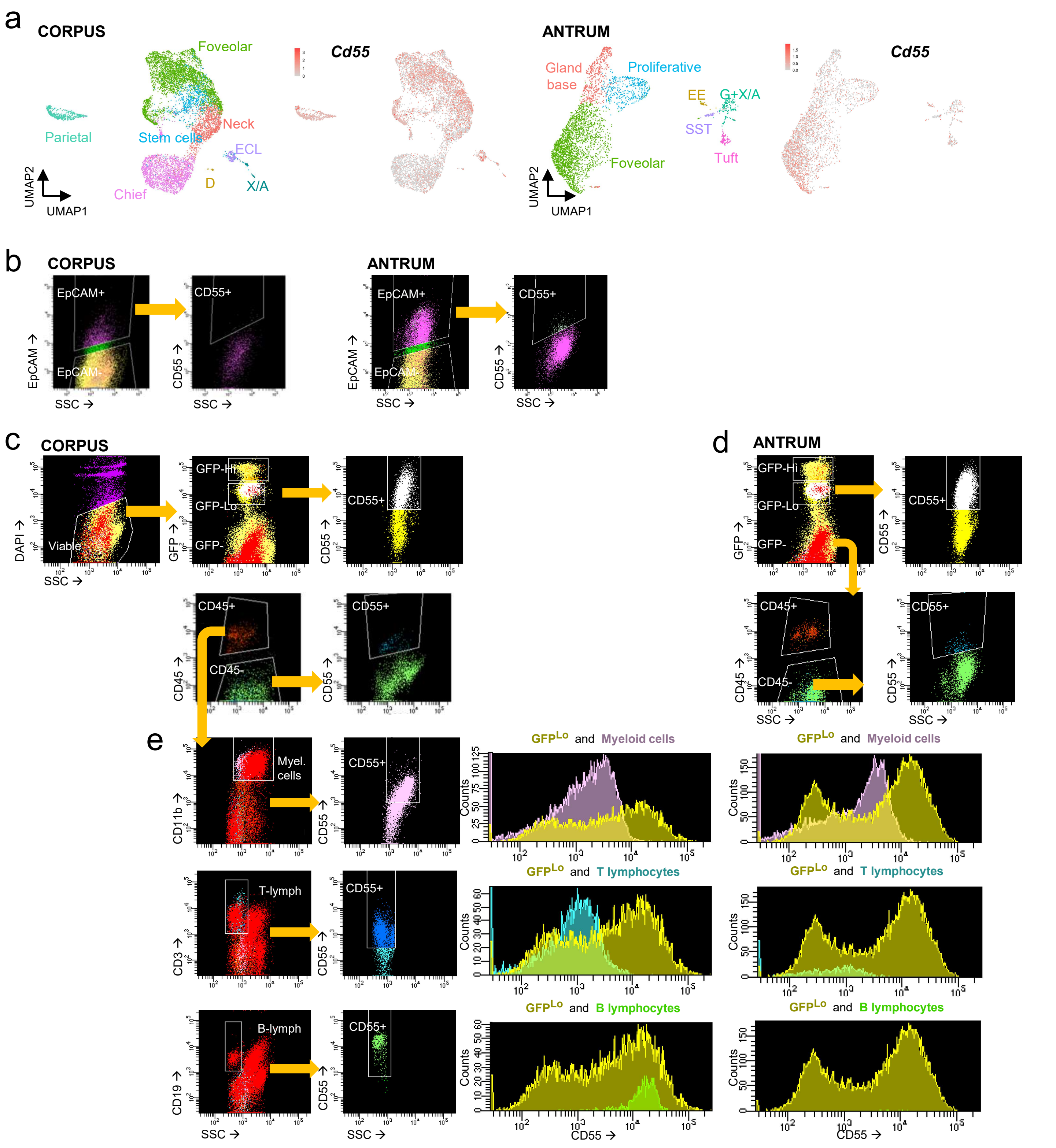
a
CORPUS
Foveolar
Parietal
Stem cells
Neck
ECL
Chief
D
X/A
UMAP2
UMAP1
Cd55
ANTRUM
Gland base
Proliferative
EE
G+X/A
SST
Tuft
Foveolar
b
CORPUS
EpCAM+
EpCAM-
CD55+
EpCAM →
SSC →
CD55 →
ANTRUM
c
CORPUS
Viable
DAPI →
GFP-Hi
GFP-Lo
GFP-
GFP →
CD55+
CD45+
CD45-
CD45 →
d
ANTRUM
e
Myel. cells
CD11b →
T-lymph
CD3 →
B-lymph
CD19 →
GFPLo and Myeloid cells
GFPLo and T lymphocytes
GFPLo and B lymphocytes
Counts
CD55 →

Supplementary Figure 7. CD55 expression in epithelial, mesenchymal PDGFRA^{neg} and PDGFRA^{Lo}, and immune cells.

(a) UMAP clustering and *Cd55* gene expression analysis in single corpus (left, n=7,637 cells combined from 3 published datasets[14,21,86]) and antrum (right, this study – see Suppl. Fig. 6, n=3,885) epithelial cells. *Cd55* transcripts are present, especially in foveolar epithelial cells.

(b) Flow cytometry for EpCAM identifies corpus (left) and antral (right) epithelial cells, which lack surface CD55 expression.

(c,d) Flow cytometry for GFP separates whole corpus (a) or antrum (b) mesenchyme into PDGFRA^{Hi}, PDGFRA^{Lo}, and PDGFRA^{neg} cells, and additional staining with CD55 antibody separates PDGFRA^{Lo} cells into CD55^{+} and CD55^{-} fractions, while PDGFRA^{neg} are mainly CD55^{-}.

(e) Immune cells from corpus (left) and antrum (right) mesenchyme were identified using CD45 (PTPRC) Ab and fractioned into myeloid cells (CD11b^{+}) or T (CD3^{+}) and B (CD19^{+}) lymphocytes, to define surface CD55 levels in each cell population. Histograms on the right reveal that only B lymphocytes express CD55 at levels approaching those observed in PDGFRA^{Lo} CD55^{+} cells. The gating strategy is reported in Supplementary Fig. 10.

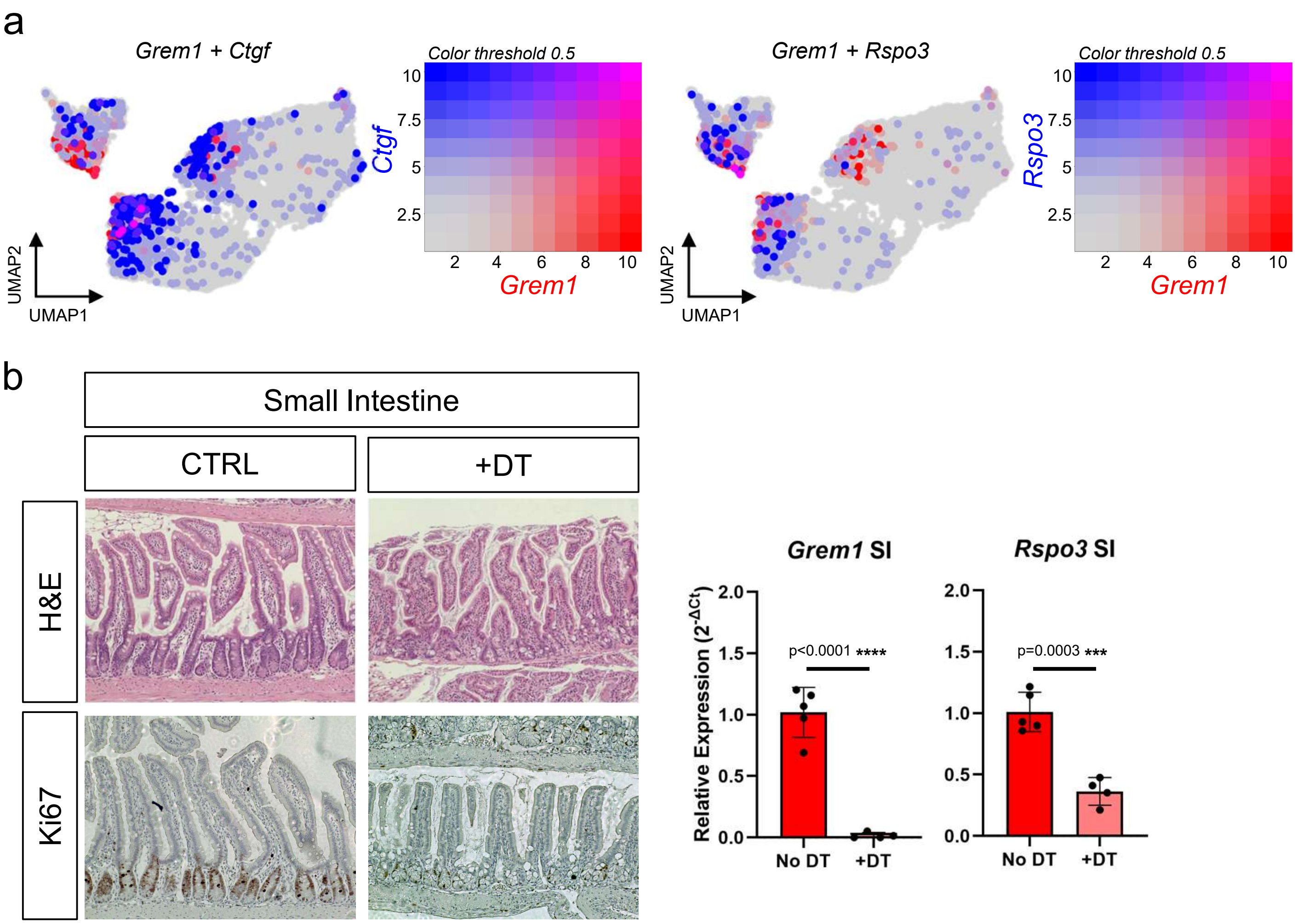
a
Grem1 + Ctgf
Color threshold 0.5
Ctgf
Grem1
UMAP2
UMAP1
Grem1 + Rspo3
Color threshold 0.5
Rspo3
Grem1
UMAP2
UMAP1
b
Small Intestine
CTRL
+DT
H&E
Ki67
Grem1 SI
Rspo3 SI
Relative Expression (2^-ΔCt)
p<0.0001 ****
p=0.0003 ***
No DT
+DT
No DT
+DT

Supplementary Figure 8. *Grem1/Ctgf/Rspo3* expression overlap, and intestinal confirmation of *Grem1*$^{+}$ cell ablation.

(a) *Grem1* + *Ctgf*, and *Grem1* + *Rspo3* co-expression densities projected onto the UMAP plot. *Grem1* and *Ctgf* are both expressed, but rarely together, in the AntLo, Corp1, and Corp2 clusters. Despite low levels of Rspo3 expression, this gene is frequently co-expressed with *Grem1*.

(b) Grem1^{DTR} mice were used to assess the effect of CD55^{+} PDGFRA^{Lo} partial depletion. SI tissue was used as a control of depletion effectiveness. Left: histology (H&E) and KI67 immunostaining confirm previously observed SI disrupted epithelial morphology and unpaired TA proliferation. Right: qRT-PCR analysis of SI tissue (relative to *Gapdh* and to No DT controls, n=5) in tissue isolated upon DT treatment. Diphtheria toxin (+DT, n=4) treatment significantly reduced *Grem1* and *Rspo3* expression in SI. Bars represent mean ±SEM values. Significance of differences was determined by two-tailed unpaired Student's t-test statistical analysis. Source data are provided as a Source Data file.

a

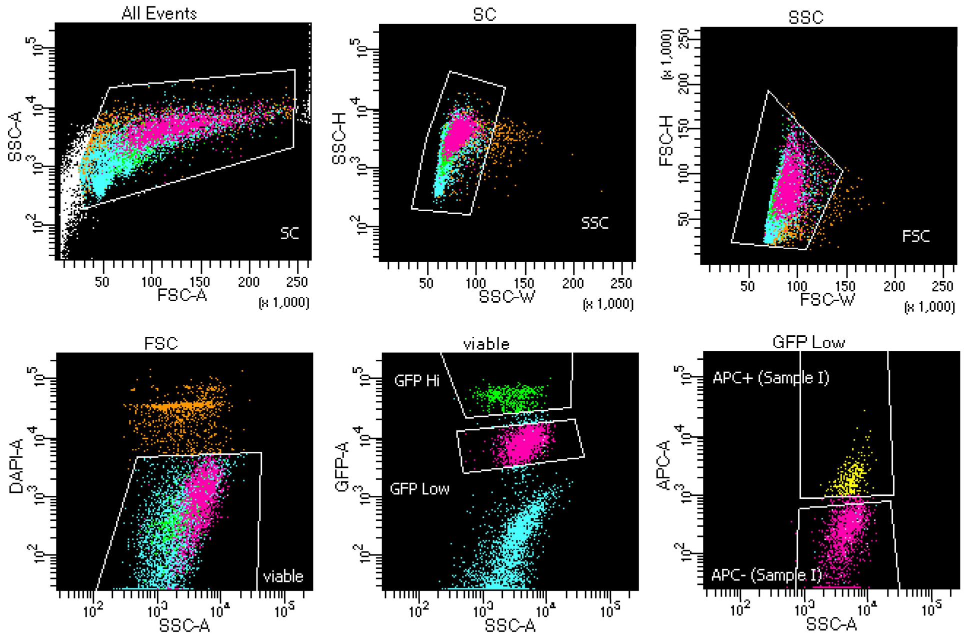
All Events
SC
SSC
FSC
viable
GFP Low
SSC-A
FSC-A
SSC-H
SSC-W
FSC-H
FSC-W
DAPI-A
GFP-A
APC-A
(x 1,000)
GFP Hi
APC+ (Sample I)
APC- (Sample I)

b

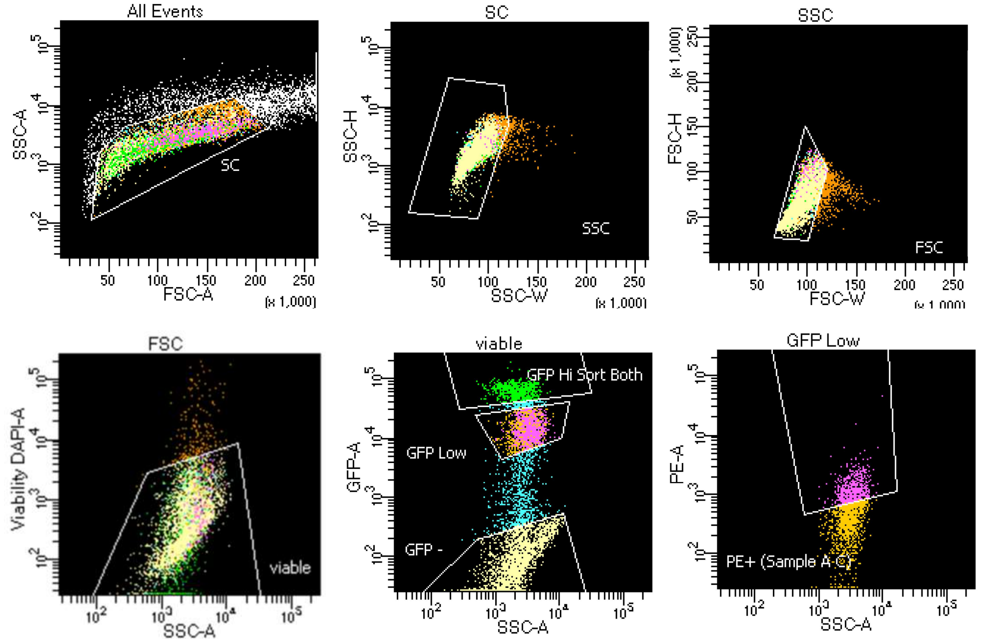
All Events
SC
SSC
FSC
viable
GFP Low
SSC-A
FSC-A
SSC-H
SSC-W
FSC-H
FSC-W
Viability DAPI-A
GFP-A
PE-A
(x 1,000)
GFP Hi Sort Both
GFP -
PE+ (Sample A-C)

c

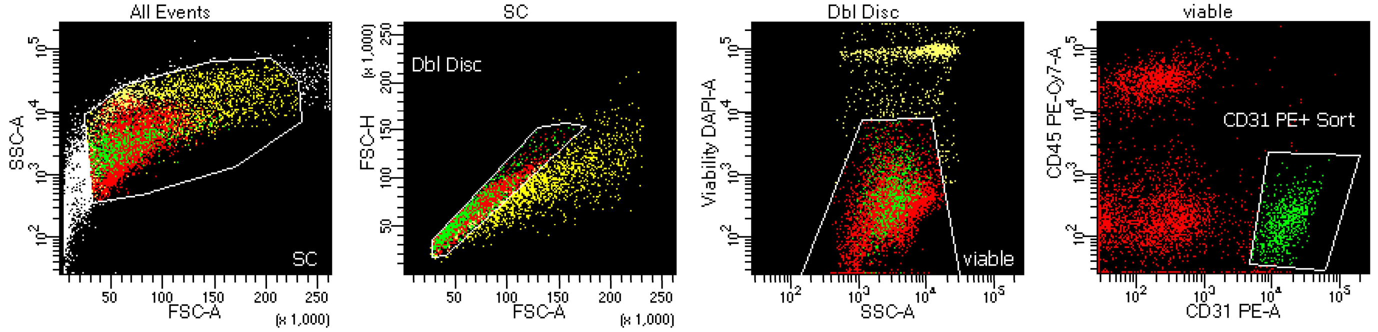
All Events
SC
Dbl Disc
viable
SSC-A
FSC-A
FSC-H
Viability DAPI-A
CD45 PE-Cy7-A
CD31 PE-A
CD31 PE+ Sort
(x 1,000)

Supplementary Figures 9. Gating strategy for co-culture experiments.

(a) Gating strategy to isolate small intestinal (SI) $PDGFRA^{Lo}$ $CD81^{+}$ cells from whole mesenchyme for co-culture experiments.

(b) Gating strategy for gastric $PDGFRA^{Hi}$, $PDGFRA^{Lo}$, $PDGFRA^{neg}$ cells isolation from whole mesenchyme for co-culture experiments. Data from corpus mesenchyme analysis are reported as an example of gating.

(c) Gating strategy for gastric $CD31^{+}$ cells isolation from whole mesenchyme for co-culture experiments. Data from corpus mesenchyme analysis are reported as an example of gating.

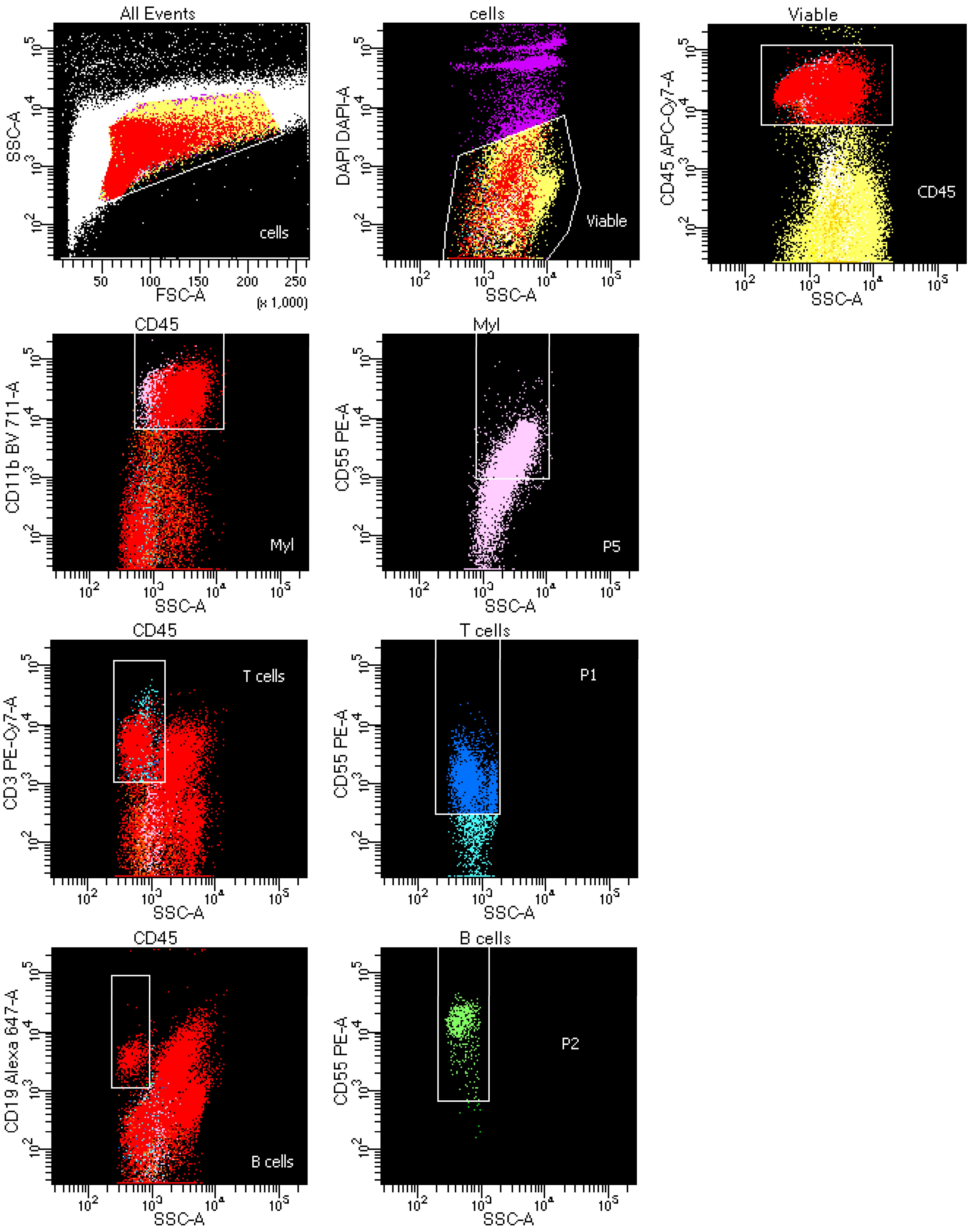
All Events
cells
FSC-A
SSC-A
(x 1,000)
cells
DAPI DAPI-A
Viable
Viable
CD45 APC-Cy7-A
CD45
CD45
CD11b BV 711-A
Myl
Myl
CD55 PE-A
P5
CD45
CD3 PE-Cy7-A
T cells
T cells
P1
CD45
CD19 Alexa 647-A
B cells
B cells
P2

Supplementary Figures 10. Gating strategy for immune cell analysis.

Cells were stained with a mixture of antibodies to identify the major immune cell populations to assess CD55 expression. CD45 was used to identify leucocytes, CD11b myeloid cells, CD3 T lymphocytes and CD19 B lymphocytes. Data from corpus mesenchyme analysis are reported as an example.

Supplementary Table 1- Oligonucleotides for *Fgf7* floxed mouse model generation and genotyping.

Primer	Sequence (5' → 3')
Fgf7_sg1_IVT_Fw	GAAATTAATACGACTCACTATAGGTATATCATTACTCACGAATCGTTTTAGAGCTAGAAATAGCAAGTTAAAATAAGGCTAGTCCG
Fgf7_sg2_IVT_Fw	GAAATTAATACGACTCACTATAGGGCAGATAATTTTGATAGAGTGTTTTAGAGCTAGAAATAGCAAGTTAAAATAAGGCTAGTCCG
IVT_Rv	AAAAAAGCACCGACTCGGTGCCACTTTTTCAAGTTGATAACGGACTAGCCTTATTTTAACTTGC
Fgf7_ex2_Fw	GGAGGGATACTTTTATGTTATGAGTTCAG (genotyping)
Fgf7_ex2_Rv	AATAGTTTGCCTTAGTGCTGGAG (genotyping)

Supplementary Table 2- Oligonucleotides for gene expression quantitation.

Gene	Forward primer	Reverse primer
Atp4a	GATGGAGATTAACGACCACCAG	ACGGGCAAACTTCACATACTC
Atp4b	CAGGAGAAGAAGTCATGCAGC	GAAACCTGCGTAGTACAGGCT
Axin2	TGACTCTCCTTCCAGATCCCA	TGCCCACACTAGGCTGAC
Cd44	TGCCTCAACTGTGCACTCAA	GTTCTGGGCTTCTTGCCTCT
Chga	ATCCTCTCTATCCTGCGACAC	GGGCTCTGGTTCTCAAACACT
Chgb	GCTCAGCTCCAGTGGATAACA	CAGGGGTGATCGTTGGAACAC
Gapdh	TCACCATCTTCCAGGAGCG	AAGCAGTTGGTGGTGCAGG
Gast	AGGGGACACCAAGGTGATGA	AGCAGATTCTGGTGTCGCAG
Gif	AAGCACAGCGCAAAAACTCC	GCAACCCCTTCATCCAAAGG
Pgc	ATGAAGAGTATCCGGGAGACC	TGGGCTCATAGAGTACACTGTAG
Tnfrsf19 (TROY)	TCTCCTAGTTCGCCTGCCTT	AAGAGCACCGTCCTGTGTAG
Grem1	CGCTCTCCTTCGTCTTCCT	CGATTCATTCTGTCACTTCCGT
Rspo3	CGAAGAATGCATCCTAATGTCAG	TATCCACTTGGACACGAAGAG
Stmn1	ACAACAACTTCAGCAAGATGG	TGTCAGGAGGGAAAGTAAAGG
