# Supplementary material for: Role of PDGFRA+ cells and a CD55+ PDGFRALo fraction in the gastric mesenchymal niche
Source: Nat Commun. 2023 Dec 2;14:7978. doi: 10.1038/s41467-023-43619-y (PMC10693581; doi:10.1038/s41467-023-43619-y)
Supplement: Supplementary file 3 — Description of Additional Supplementary Files [file 41467_2023_43619_MOESM3_ESM.pdf]

Supplementary Data 1. Differentially expressed genes across bulk RNAseq samples.
Bulk RNA-seq data were analyzed with DESeq2 (p_{adj} <0.05; |$\log_2$ fold-change| >1.5). Default settings were used for statistical analysis (Wald test).

Supplementary Data 2. scRNAseq PDGFRA$^+$ population markers.
scRNA-seq data from PDGFRA$^+$ cells were analyzed using Seurat FindMarkers tool with default settings (Wilcoxon rank sum test, logfc treshold 0.25).

Supplementary Data 3. scRNAseq of foveolar cells population isolated from WT and *Fgf7* KO mice.
scRNA-seq data from PDGFRA+ cells were analyzed using Seurat FindMarkers tool with default settings (Wilcoxon rank sum test, logfc treshold 0.25).

Supplementary Movie 1. 3D video rendering of murine corpus blood vessels (CD31 in red).

Supplementary Movie 2. 3D video rendering of murine antral blood vessels (CD31 in red).

Supplementary Movie 3. 3D video rendering of murine corpus muscle fibers (SMA1 in yellow).

Supplementary Movie 4. 3D video rendering of murine antral muscle fibers (SMA1 in yellow).

Supplementary Movie 5. 3D video rendering of $Pdgfra^{Cre(ER\text{-}T2)}$;$Rosa26^{mT/mG}$ mouse (stained green after tamoxifen treatment) corpus to show the morphology of $Pdgfra^{Hi}$ cells enveloping each gland and additional $Pdgfra^{Lo}$ cells lying in the space beneath glands.

Supplementary Movie 6. 3D video rendering of $Pdgfra^{Cre(ER\text{-}T2)}$;$Rosa26^{mT/mG}$ mouse (stained green after tamoxifen treatment) antrum to show the morphology of $Pdgfra^{Hi}$ cells enveloping each gland and additional $Pdgfra^{Lo}$ cells lying in the space beneath glands.
