# Supplementary material for: Role of PDGFRA+ cells and a CD55+ PDGFRALo fraction in the gastric mesenchymal niche
Source: Nat Commun. 2023 Dec 2;14:7978. doi: 10.1038/s41467-023-43619-y (PMC10693581; doi:10.1038/s41467-023-43619-y)
Supplement: Supplementary file 13 — Reporting Summary [file 41467_2023_43619_MOESM13_ESM.pdf]

nature portfolio

Corresponding author(s): Ramesh A. Shivdasani

Last updated by author(s): 10-24-2023

Reporting Summary

Nature Portfolio wishes to improve the reproducibility of the work that we publish. This form provides structure for consistency and transparency in reporting. For further information on Nature Portfolio policies, see our Editorial Policies and the Editorial Policy Checklist.

Statistics

For all statistical analyses, confirm that the following items are present in the figure legend, table legend, main text, or Methods section.

n/a	Confirmed	
☐	☒	The exact sample size (*n*) for each experimental group/condition, given as a discrete number and unit of measurement
☐	☒	A statement on whether measurements were taken from distinct samples or whether the same sample was measured repeatedly
☐	☒	The statistical test(s) used AND whether they are one- or two-sided<br>*Only common tests should be described solely by name; describe more complex techniques in the Methods section.*
☒	☐	A description of all covariates tested
☐	☒	A description of any assumptions or corrections, such as tests of normality and adjustment for multiple comparisons
☐	☒	A full description of the statistical parameters including central tendency (e.g. means) or other basic estimates (e.g. regression coefficient) AND variation (e.g. standard deviation) or associated estimates of uncertainty (e.g. confidence intervals)
☐	☒	For null hypothesis testing, the test statistic (e.g. *F*, *t*, *r*) with confidence intervals, effect sizes, degrees of freedom and *P* value noted<br>*Give P values as exact values whenever suitable.*
☒	☐	For Bayesian analysis, information on the choice of priors and Markov chain Monte Carlo settings
☐	☒	For hierarchical and complex designs, identification of the appropriate level for tests and full reporting of outcomes
☐	☒	Estimates of effect sizes (e.g. Cohen's *d*, Pearson's *r*), indicating how they were calculated

Our web collection on statistics for biologists contains articles on many of the points above.

Software and code

Policy information about availability of computer code

Data collection	- LightCycler 480 instrument (Roche) for quantitative PCR analysis<br>- Cells were sorted on a BD FACSAria II cell sorter with BD FACSDiva 8.0.1 software<br>- Data from cells were collected by FACS on a BD LSR Fortessa cell analyzer<br>- Images were taken using an SP5X laser scanning confocal microscope (Leica Microsystems CMS GmbH) or an LSM980 microscope and ZEN Desk 3.4 software (Carl Zeiss GmbH).<br>- Bulk and single cellRNAseq libraries were sequenced using a NovaSeq platform (Illumina).
Data analysis	- Images were analyzed using Fiji and ZEN Desk 3.4 software.<br>- Videos were generated with ZEN Desk 3.4 software.<br>- GFP+ cell images were quantified using Cellpose 2.0 plugin in CellProfiler 4.2.1<br>- GFP signal was quantified using Cellpose 2.0 and feeding segmented masks to CellProfiler 4.2.1<br>- GraphPad Prism version 9<br>- R platform 4.2.1<br>- Bulk RNAseq analysis: Data were aligned to mouse reference genome mm10 using the Viper pipeline with default settings. Data quality was verified using RSeQC. Data were normalized and differential gene expression (padj <0.05; |log2 fold-change| >1.5) was analyzed with the DESeq2 package. Pearson correlation coefficients were calculated from DESeq2 normalized counts and plotted using Corrplot package. Integrative Genome Viewer (IGV) data tracks were generated from RPKM-normalized bigwigs loaded into IGV 2.15.4 (Broad Institute).<br>- scRNAseq analysis: Samples were aligned to the mm10 genome using 10x Genomics Cell Ranger 3.0.2 with default parameters. Single-cell analyses used Seurat 4.1.0. Cell features were detected with the FindVariableFeatures function (2,000 features, selection method “vst”). The first 15 principal components were taken to run uniform manifold approximation and projection (UMAP) algorithms. Cell types were identified

using established marker genes detected by the "FindAllMarkers" function in Seurat2 (min.pct=0.25, test "roc").

For manuscripts utilizing custom algorithms or software that are central to the research but not yet described in published literature, software must be made available to editors and reviewers. We strongly encourage code deposition in a community repository (e.g. GitHub). See the Nature Portfolio guidelines for submitting code & software for further information.

Data

Policy information about availability of data

All manuscripts must include a data availability statement. This statement should provide the following information, where applicable:

- Accession codes, unique identifiers, or web links for publicly available datasets
- A description of any restrictions on data availability
- For clinical datasets or third party data, please ensure that the statement adheres to our policy

- mouse reference genome mm10
- Sequencing data generated in this study is available on NCBI GEO under the accession numbers GSE224737
- Dataset reanalyzed here can be found at NCBI GEO under the accession number GSE130681 (McCarthy et al., Cell Stem Cell 2020)
- This study includes analysis of previously published data, ArrayExpress accession numbers E-MTAB-6879 and E-MTAB-11597, and NCBI Sequence Read Archive SRP227356.

Research involving human participants, their data, or biological material

Policy information about studies with human participants or human data. See also policy information about sex, gender (identity/presentation), and sexual orientation and race, ethnicity and racism.

Reporting on sex and gender	*Use the terms sex (biological attribute) and gender (shaped by social and cultural circumstances) carefully in order to avoid confusing both terms. Indicate if findings apply to only one sex or gender; describe whether sex and gender were considered in study design; whether sex and/or gender was determined based on self-reporting or assigned and methods used. Provide in the source data disaggregated sex and gender data, where this information has been collected, and if consent has been obtained for sharing of individual-level data; provide overall numbers in this Reporting Summary. Please state if this information has not been collected. Report sex- and gender-based analyses where performed, justify reasons for lack of sex- and gender-based analysis.*
Reporting on race, ethnicity, or other socially relevant groupings	*Please specify the socially constructed or socially relevant categorization variable(s) used in your manuscript and explain why they were used. Please note that such variables should not be used as proxies for other socially constructed/relevant variables (for example, race or ethnicity should not be used as a proxy for socioeconomic status). Provide clear definitions of the relevant terms used, how they were provided (by the participants/respondents, the researchers, or third parties), and the method(s) used to classify people into the different categories (e.g. self-report, census or administrative data, social media data, etc.) Please provide details about how you controlled for confounding variables in your analyses.*
Population characteristics	*Describe the covariate-relevant population characteristics of the human research participants (e.g. age, genotypic information, past and current diagnosis and treatment categories). If you filled out the behavioural & social sciences study design questions and have nothing to add here, write "See above."*
Recruitment	*Describe how participants were recruited. Outline any potential self-selection bias or other biases that may be present and how these are likely to impact results.*
Ethics oversight	*Identify the organization(s) that approved the study protocol.*

Note that full information on the approval of the study protocol must also be provided in the manuscript.

Field-specific reporting

Please select the one below that is the best fit for your research. If you are not sure, read the appropriate sections before making your selection.

- [x] Life sciences
- [ ] Behavioural & social sciences
- [ ] Ecological, evolutionary & environmental sciences

For a reference copy of the document with all sections, see nature.com/documents/nr-reporting-summary-flat.pdf

Life sciences study design

All studies must disclose on these points even when the disclosure is negative.

Sample size	No statistical methods were used to determine sample size
Data exclusions	- Reads from genes known to express in gastric epithelium and genes encoded on sex chromosomes were excluded from Bulk RNAseq analysis<br>- For scRNAseq analysis, cells were filtered for <12% mitochondrial and >1,000 unique reads.

Replication	Biological replicates were performed at least 3 times. All attempts at replication were successful.<br>The mean +/- S.E.M, and statistical analysis (ANOVA Tukey's multiple comparison test, one-way ANOVA Dunnett's multiple comparison test, unpaired two-tailed t-test) was used to see if and how the difference between parameters are significant. Significance were presented as stars and p values, calculated by Graph Pad software.
Randomization	Samples were not randomized for this study
Blinding	There was no blinded allocation of samples during experiments or analysis

Reporting for specific materials, systems and methods

We require information from authors about some types of materials, experimental systems and methods used in many studies. Here, indicate whether each material, system or method listed is relevant to your study. If you are not sure if a list item applies to your research, read the appropriate section before selecting a response.

Materials & experimental systems

n/a	Involved in the study
☐	☒ Antibodies
☒	☐ Eukaryotic cell lines
☒	☐ Palaeontology and archaeology
☐	☒ Animals and other organisms
☒	☐ Clinical data
☒	☐ Dual use research of concern
☒	☐ Plants

Methods

n/a	Involved in the study
☒	☐ ChIP-seq
☐	☒ Flow cytometry
☒	☐ MRI-based neuroimaging

Antibodies

Antibodies used

CD31 (BD Cell Analysis, 557355);
Laminin (Sigma Aldrich, L9393);
alpha-smooth muscle actin1 (SMA1, Abcam, ab5694);
PDGFRA (R&D Systems, AF1062, 1:100);
MUC5AC (Cell Signaling Technology, 6119S);
Gastrin (Abcam, ab232775, 1:100);
Somatostatin (Santa Cruz Biotechnology, sc-55565, 1:100);
biotin-CD81 antibody (eBioscience, 13081181, 1:100);
streptavidin-APC conjugated secondary antibody (eBioscience, 17431782, 1:100);
PE-conjugated CD55 antibody (BioLegend, 131803, 1:75);
AlexaFluor Goat 546 anti-rat IgG (Invitrogen, A11081)
AlexaFluor Goat 633 anti-rabbit IgG (Invitrogen, A21071)
AlexaFluor Donkey 594 anti-goat IgG (Invitrogen, A11058)
APC-conjugated EpCAM (BioLegend, 118214, 1:100)
PE-conjugated CD31 (BD Biosciences, 553373)
PE-Cy7-conjugated CD45 (Invitrogen, 25-0451-82)
TruStain FcX PLUS (BioLegend, 156603)
BV711 anti-CD11b (BD Biosciences, 563168);
Alexa Fluor 647 anti-CD19 (BioLegend, 115525);
PE/Cy7 anti-CD3 (BioLegend, 100219);
DAPI (BD Biosciences, 564907)

Validation

- CD31 (BD Cell Analysis, 557355); https://www.bdbiosciences.com/en-us/products/reagents/flow-cytometry-reagents/research-reagents/single-color-antibodies-ruo/purified-rat-anti-mouse-cd31.557355
- Laminin (Sigma Aldrich, L9393); https://www.sigmaaldrich.com/US/en/product/sigma/l9393?gclid=Cj0KCQiA0oagBhDHARIsAI-BbgeSnsc9iUTzpsC3-RHfQGQtGa5ueI8f8YGCTDyE2OEHtv3evgVAOUcaAqobEALw_wcB&gclsrc=aw.ds
- alpha-smooth muscle actin1 (SMA1, Abcam, ab5694); https://www.abcam.com/products/primary-antibodies/alpha-smooth-muscle-actin-antibody-ab5694.html
- PDGFRA (R&D Systems, AF1062); https://www.rndsystems.com/products/mouse-pdgf-ralpha-antibody_af1062
- MUC5AC (Cell Signaling Technology, 61193); https://www.cellsignal.com/products/primary-antibodies/muc5ac-e3o9i-xp-rabbit-mab/61193?site-search-type=Products&N=4294956287&Ntt=muc5ac&fromPage=plp
- Gastrin (Abcam, ab232775); https://www.abcam.com/products/primary-antibodies/gastrin-antibody-ab232775.html
- Somatostatin (Santa Cruz Biotechnology, sc-55565); https://www.scbt.com/p/somatostatin-antibody-g-10?gclid=Cj0KCQiA0oagBhDHARIsAI-Bbgd5U0b04A5HtCRYuwXSsODkKV0tPz4Gx1v2eOZoVQGkzWQMVddcib0aAvUnEALw_wcB
- biotin-CD81 antibody (eBioscience, 13081181); https://www.fishersci.com/shop/products/cd81-armenian-hamster-anti-mouse-biotin-clone-eat-2-eat2-ebioscience/5011640
- streptavidin-APC conjugated secondary antibody (eBioscience, 17431782); https://www.thermofisher.com/order/catalog/

product/17-4317-82?gclid=Cj0KCQiA0oagBhDHARIsAI-BbgdP8GLhHka7oTvrPbfsONitfMoil-o2iUQ1zD5v4lT-K5S548R-oe0aAvHyEALw_wcB&ef_id=Cj0KCQiA0oagBhDHARIsAI-BbgdP8GLhHka7oTvrPbfsONitfMoil-o2iUQ1zD5v4lT-K5S548R-oe0aAvHyEALw_wcB:G:s&s_kwcid=AL!3652!3!278870232429!!!g!!!1454324556!63404918784&cid=bid_pca_frg_r01_co_cp1359_pjt0000_bid00000_0se_gaw_dy_pur_con
- PE-conjugated CD55 antibody (BioLegend, 131803); https://www.biolegend.com/fr-ch/products/pe-anti-mouse-cd55-daf-antibody-5514
- AlexaFluor Goat 546 anti-rat IgG (Invitrogen, A11081); https://www.thermofisher.com/antibody/product/Goat-anti-Rat-IgG-H-L-Cross-Adsorbed-Secondary-Antibody-Polyclonal/A-11081
- AlexaFluor Goat 633 anti-rabbit IgG (Invitrogen, A21071); https://www.thermofisher.com/antibody/product/Goat-anti-Rabbit-IgG-H-L-Highly-Cross-Adsorbed-Secondary-Antibody-Polyclonal/A-21071
- AlexaFluor Donkey 594 anti-goat IgG (Invitrogen, A11058); https://www.thermofisher.com/antibody/product/Donkey-anti-Goat-IgG-H-L-Cross-Adsorbed-Secondary-Antibody-Polyclonal/A-11058
- APC-conjugated EpCAM (BioLegend, 118214); https://www.biolegend.com/de-at/products/apc-anti-mouse-cd326-ep-cam-antibody-4974
- PE-conjugated CD31 antibody (BD Biosciences, 553373); https://www.bdbiosciences.com/en-us/products/reagents/flow-cytometry-reagents/research-reagents/single-color-antibodies-ruo/pe-rat-anti-mouse-cd31.553373
- PE-Cy7-conjugated CD45 antibody (Invitrogen, 25-0451-82); https://www.thermofisher.com/antibody/product/CD45-Antibody-clone-30-F11-Monoclonal/25-0451-82
- BV711 anti-CD11b (BD Biosciences, 563168); https://www.bdbiosciences.com/en-us/products/reagents/flow-cytometry-reagents/research-reagents/single-color-antibodies-ruo/bv711-rat-anti-cd11b.563168
- Alexa Fluor 647 anti-CD19 (BioLegend, 115525); https://www.biolegend.com/en-us/products/alexa-fluor-647-anti-mouse-cd19-antibody-2705
- PE/Cy7 anti-CD3 (BioLegend, 100219); https://www.biolegend.com/en-us/products/pe-cyanine7-anti-mouse-cd3-antibody-6060
- DAPI (BD Biosciences, 564907); https://www.bdbiosciences.com/en-us/products/reagents/flow-cytometry-reagents/research-reagents/single-color-antibodies-ruo/dapi-solution.564907

Animals and other research organisms

Policy information about studies involving animals; ARRIVE guidelines recommended for reporting animal research, and Sex and Gender in Research

Laboratory animals

Mice were used in this study. Mice were housed in a pathogen-free animal facility and maintained on a 12-h light/dark cycle at constant temperature and humidity, with ad libitum access to food and water.
Strains were maintained on a mixed C57BL/6 background. Male and females aged 8-16 weeks were used.
- PdgfraH2BeGFP (JAX strain 007669)
- PdgfraCre(ER-T2) (JAX strain 032770)
- Rosa26Lsl-TdTomato (JAX strain 007908)
- Rosa26mT/mG (JAX strain 07676)
- Rosa26mT/mG mice were crossed with PdgfraCre(ER-T2) to generate compound heterozygotes.
- Fgf7fl/fl mice were generated in this study and crossed with PdgfraCre(ER-T2) and Rosa26Lsl-TdTomato to obtain PdgfraCre(ER-T2);Fgf7fl/fl;Rosa26LSL-TdTomato mice
- Grem1-P2A-DTR-P2A-TdTomato (Grem1DTR) mice were described previously (Kraiczy et al.)

Wild animals

No wild animals were used in this study

Reporting on sex

Sex was not considered, males and females were used for this study

Field-collected samples

No field-collected samples were used in this study

Ethics oversight

All animal procedures were approved by Animal Care and Use Committees at the Dana-Farber Cancer Institute or Columbia University Medical Center.

Note that full information on the approval of the study protocol must also be provided in the manuscript.

Plants

Seed stocks

Report on the source of all seed stocks or other plant material used. If applicable, state the seed stock centre and catalogue number. If plant specimens were collected from the field, describe the collection location, date and sampling procedures.

Novel plant genotypes

Describe the methods by which all novel plant genotypes were produced. This includes those generated by transgenic approaches, gene editing, chemical/radiation-based mutagenesis and hybridization. For transgenic lines, describe the transformation method, the number of independent lines analyzed and the generation upon which experiments were performed. For gene-edited lines, describe the editor used, the endogenous sequence targeted for editing, the targeting guide RNA sequence (if applicable) and how the editor was applied.

Authentication

Describe any authentication procedures for each seed stock used or novel genotype generated. Describe any experiments used to assess the effect of a mutation and, where applicable, how potential secondary effects (e.g. second site T-DNA insertions, mosiacism, off-target gene editing) were examined.

Flow Cytometry

Plots

Confirm that:

- [x] The axis labels state the marker and fluorochrome used (e.g. CD4-FITC).
- [x] The axis scales are clearly visible. Include numbers along axes only for bottom left plot of group (a 'group' is an analysis of identical markers).
- [ ] All plots are contour plots with outliers or pseudocolor plots.
- [ ] A numerical value for number of cells or percentage (with statistics) is provided.

Methodology

Sample preparation	Small intestine, antral, and corpus mesenchyme was isolated as follow: tissue was harvested after perfusing adult Pdgfra-H2BeGFP mice with cold PBS. After manual removal of external muscles, the epithelium was removed by rotating the tissue at 250 rpm in pre-warmed chelation buffer (10 mM EDTA, 5% FBS, 1 mM Dithiothreitol-DTT, 10 mM HEPES in Hank's Balanced Salt Solution, HBSS) for 20 min at 37°C. The tissue was then washed with PBS and digested for 10 min at 37°C in 3 mg/ml collagenase II (Worthington, LS004176) in HBSS supplemented with 5% FBS and 10 mM HEPES. Cells were harvested by centrifugation at 300g for 5 min. For bulk RNA sequencing, cells were sorted on a BD FACSAria II cell sorter, gating on DAPI negative cells to identify live cells, and GFP to sort Pdgfra-high or -low expressing cells. All DAPI-negative viable cells were collected for single cell-RNA sequencing.<br>- Co-culture experiments: whole mesenchyme was isolated and plated on tissue culture plates (small intestine) or fibronectin-coated tissue culture plates (gastric mesenchyme, Corning, 354451) in DMEM/F12 medium supplemented with 10% FBS, 1X penicillin/streptomycin, 1X normocin, 1X primocin, 1X Glutamax, and 10 mM HEPES. Three days later, cells were washed in PBS, removed from the plates with 0.25% Trypsin (5 min at 37°C), washed, and stained with antibodies. Small intestine mesenchyme was stained with biotin-CD81 antibody (eBioscience, 13081181, 1:100) followed by incubation with streptavidin-APC conjugated secondary antibody (eBioscience, 17431782, 1:100). Gastric cells were incubated with PE-conjugated CD55 antibody (BioLegend, 131803, 1:75).
Instrument	BD FACSAria II cell sorter
Software	BD FACSDiva 8.0.1
Cell population abundance	Stomach: ~10% PdgfraHi cells, ~20% PdgfraLo cells from whole mesenchyme culture. ~20-30% of total PdgfraLo cells were CD55+<br>Small intestine: ~5% PdgfraHi cells, ~20% PdgfraLo cells from whole mesenchyme cultures. Of this PdgfraLo cells, approximately 45% were CD81+
Gating strategy	Cells were first gated based on similar light scatter properties, doublets gated out using the width parameter on forward scatter, and then live cells gated based on DAPI. For conjugated Ab cell surface marker staining, negative cells were used as gating parameter.

- [x] Tick this box to confirm that a figure exemplifying the gating strategy is provided in the Supplementary Information.
